# Supplementary material for: Adequacy of endoscopic recognition and surveillance of gastric intestinal metaplasia and atrophic gastritis: A multicentre retrospective study in low incidence countries
Source: PLoS One. 2023 Jun 23;18(6):e0287587. doi: 10.1371/journal.pone.0287587 (PMC10289343; doi:10.1371/journal.pone.0287587)
Supplement: S1 Table — GA: gastric atrophy GIM: gastric intestinal metaplasia. (DOCX) [file pone.0287587.s001.docx]

**S1 table Endoscopic recognition of different histopathologic conditions**

| Endoscopic diagnosis | Pathology | All centers | Center 1 | Center 2 | Center 3 |
| --- | --- | --- | --- | --- | --- |
| No endoscopic suspicion | GA and/or GIM | 213/396 (53.8%) | 44/100 (44%) | 112/213 (52.6%) | 57/83 (68.7%) |
| GA or GIM suspected | GA and/or IM | 179/396 (42.2%) | 41/100 (41%) | 97/213 (45.5%) | 19/83 (23%) |
| GIM suspected | GIM | 55/337 (16.3%) | 1/79 (1.27%) | 44/177 (24.9%) | 10/81 (1.2%) |
| GA suspected | GA (+/-GIM) | 95/196 (48.5%) | 55/90 (61.1%) | 33/81 (40.7%) | 11/25 (44%) |
