# Supplementary material for: Adequacy of endoscopic recognition and surveillance of gastric intestinal metaplasia and atrophic gastritis: A multicentre retrospective study in low incidence countries
Source: PLoS One. 2023 Jun 23;18(6):e0287587. doi: 10.1371/journal.pone.0287587 (PMC10289343; doi:10.1371/journal.pone.0287587)
Supplement: S2 Table — Center 1: Adequate surveillance in 23 of 100 patients (23%) or 54 (54%) when including the cases without surveillance because of age. Center 2: Adequate surveillance in 82 of 212 patients (38,5%) and 118 (55,4%) when including the cases without surveillance because of age. Center 3: Adequate surveillance in 41 of 83 patients (49,4%) and 43 (51,8%) when including the cases without surveillance because of age. (DOCX) [file pone.0287587.s002.docx]

**S2 table. Adequacy of surveillance in different centers**

| Center 1  (N=100) | Adequate surveillance | Inadequate  surveillance | No surveillance as per guidelines | No surveillance due to age | Total |
| --- | --- | --- | --- | --- | --- |
| No GIM | 2 | 12 | 0 | 7 | 21 |
| Proximal GIM | 8 | 13 | 0 | 6 | 27 |
| Distal GIM | 2 | 3 | 3 | 3 | 11 |
| Pangastric GIM | 5 | 8 | 0 | 6 | 19 |
| GIM unknown location | 2 | 10 | 0 | 9 | 22 |
| Total | 20 | 46 | 3 | 31 |  |
| Center 2 (N=213) |  |  |  |  |  |
| No GIM | 6 | 22 | 1 | 7 | 36 |
| Proximal GIM | 5 | 20 | 0 | 3 | 28 |
| Distal GIM | 9 | 19 | 51 | 11 | 90 |
| Pangastric GIM | 8 | 25 | 1 | 12 | 46 |
| GIM unknown location | 1 | 9 | 0 | 3 | 13 |
| Total | 29 | 95 | 53 | 36 |  |
| Center 3  (N=83) |  |  |  |  |  |
| No GIM | 0 | 2 | 0 | 0 | 2 |
| Proximal GIM | 1 | 13 | 3 | 1 | 18 |
| Distal GIM | 4 | 12 | 27 | 1 | 44 |
| Pangastric GIM | 3 | 10 | 2 | 0 | 15 |
| GIM unknown location | 0 | 3 | 1 | 0 | 4 |
| Total | 8 | 40 | 33 | 2 |  |
