# Supplementary material for: Adequacy of endoscopic recognition and surveillance of gastric intestinal metaplasia and atrophic gastritis: A multicentre retrospective study in low incidence countries
Source: PLoS One. 2023 Jun 23;18(6):e0287587. doi: 10.1371/journal.pone.0287587 (PMC10289343; doi:10.1371/journal.pone.0287587)
Supplement: S3 Table — * refers to antrum and incisura. (DOCX) [file pone.0287587.s003.docx]

**S3 table. Distribution of the biopsies based on Sydney protocol**

|  | All (N=396) | Center 1 (N=100) | Center 2 (N=213) | Center 3  (N=83) |
| --- | --- | --- | --- | --- |
| Biopsy location   - Proximal - Distal* - Both - Unknown | 37 (9%)  58 (15%)  268 (68%)  33 (8%) | 17 (17%)  5 (5%)  49 (49%)  29 (29%) | 16 (8%)  36 (17%)  158 (74%)  3 (1%) | 4 (5%)  17 (21%)  61 (74%)  1 (1%) |

* refers to antrum and incisura
