# Supplementary material for: Adequacy of endoscopic recognition and surveillance of gastric intestinal metaplasia and atrophic gastritis: A multicentre retrospective study in low incidence countries
Source: PLoS One. 2023 Jun 23;18(6):e0287587. doi: 10.1371/journal.pone.0287587 (PMC10289343; doi:10.1371/journal.pone.0287587)
Supplement: Attachment — Submitted filename: Response to Reviewers_1.docx [file pone.0287587.s005.docx]

**Early Cancer Institute**

Cambridge Biomedical Campus

Box 197, CB20XZ

Cambridge, UK

To

Editorial board of Plos One

Date	April 14^th^, 2023

Re. manuscript resubmission

Dear Ozlem Boybeyi-Turer, dear editorial board and reviewers,

We would like to thank you for your thorough review of our manuscript with valuable comments. Please find below a point-to-point reply to the reviewers’ comments. In addition to these we have created a minimal dataset which we have added as Supporting information.

On behalf of all authors,

Yours Sincerely,

Dr.Massimiliano di Pietro

Senior clinician Scientist

Honorary Gastroenterologist

Early Cancer Insititute, Cambridge

University of Cambridge, UK

Dr.Judith Honing

Clinical Research Fellow

Early Cancer Institute, Cambridge

University of Cambridge, UK

Point to point reply

In retrieving the considered patients, the authors regarded the definitions of atrophic gastritis and intestinal metaplasia as equivalent. The two conditions are not biologically nor clinically the same, being IM one of the histological phenotypes included in the Atrophy definition.

> Thank you for pointing out that we gave the impression in our paper that these are the same. Although part of a spectrum we definitely agree these should be regarded as different entities. We have added in the introduction a sentence to refer to the Correa cascade (page 5, line 7-11). In addition, we have made textual changes in the manuscript to clarify that we do regard them as different conditions, eg instead of GA/GIM we refer to them as GA and/or GIM.

The reliability of endoscopy in atrophy detection is considered lower than that of IM (particularly by adopting high-resolution instruments). It is frankly unexpected the higher value of consistency in endoscopic detection of atrophy versus IM (48.5 % for GA versus 16.3 % for IM). This impressive result should be carefully considered and confirmed.

> We agree with the reviewer that with the current imaging-enhancing techniques one would not expect the IM rate to be much lower than the GA rate. However after reviewing all the endoscopy reports it does seem to be the case that features of GA have easier to be recognized than GIM features. We suspect this is due to the fact that loss of rugal folds and an atrophic border can be seen with white light, while IM can appear as gastritis on white light. In fact, in many of the reports gastritis was mentioned as the reason to biopsy. This suggests that in clinical practice the endoscopist does not recognize GIM and confuses GIM for an inflammatory gastritis. Using NBI to distinguish the two from each other would be beneficial but is often not done due to time constraints and lack of training. We suspect endoscopists, when recognizing GA, don’t proceed to the next step of looking for IM and lesions, regarding this as a disease of the whole stomach.

The higher GA recognition rate includes both GA cases with and without GIM, however it should be noted in cases with GIM endoscopists still did not recognize the IM. We have now clarified this in Methods/Definition (Page 8, line 4-7) and in the Figure 1 legend. In addition, we have added a supplementary table 1 which shows these numbers in more detail with the breakdown of individual endoscopic diagnoses. In particular we show that cases in cases with any histopathological pre-malignant diagnosis (GA and/or GIM) the rate of endoscopic diagnosis of an atrophic or metaplastic process was 42.2%. We have added a sentence in the results section at page 12, line 11-12.

Finally the relevance and implication of this findings has been discussed in details at (page 16, 1-9).

The definition of pangastritis includes heterogeneous clinical and histological situations. According to the Sydney system, the current (ambiguous) definitions of pangastritis includes different conditions associated with different GC risk:
(a) sparse foci of atrophy involving the mucous-secreting antrum and the oxyntic compartment;
(b) extensive atrophic lesions involving both the antrum and body (i.e., Open Type gastritis according to Kimura and Takemoto).

I agree entirely with the author's choice of assuming histology as a reference standard in atrophy assessment. Histology reliability - however - is strongly conditioned by the biopsy sampling protocol. No information about this crucial issue is reported.

- We strongly agree with the reviewer that adequate sampling is crucial to determine the disease extent. The breakdown of the biopsy data in terms of extent of the atrophic process is provided in supplementary table 2. The definitions of the different histopathological situations is provided in the Methods section and this was amended using the suggested wording (page 7, 19-23). The endoscopic biopsy definition is provided methods section in paragraph ‘Endoscopy’ (page 9 line 1-3). The corresponding results and reference are presented in the Results section (page 11, line 6-8).

Reviewer 2

The paper titled: "Adequacy of endoscopic recognition and surveillance of gastric intestinal metaplasia and atrophic gastritis: a multicentre retrospective study in low incidence countries" is very interesting regarding the rate of endoscopic recognition of gastric pre-cancerous lesions. Also, the authors state well the limitations of this study.

However, I would like to ask the authors to provide more info with regards to the histological confirmation of the cases that they studies. For all the cases did the histological analysis confirm correctly the status of each case?

- All cases were retrieved by histopathological diagnosis. We’ve used this approach to investigate the true endoscopic recognition rate using the gold standard (histopathology) as starting point. We have emphasized this in the revised manuscript in the introduction under the aims of this study: ‘the aims of this study were i. to quantify the rate of endoscopic recognition of GA and GIM in a real-life clinical practice using histopathologic diagnosis as gold standard’ (page 6, 5-7).
- We provide details of the histopathological diagnosis in the Methods section under paragraph “Definitions” (page 7, 19-23)

Also, what was the time between the histological analysis and the endoscopy. The authors should provide a comparison between endoscopic and histological confirmation of the cases (as the histological confirmation remains the gold standard).

- The biopsies were taken at the time of the endoscopy. The interval between the procedure and histopathologic report can vary but usually it was around 2-3 weeks. The management decisions were taken accordingly after report available. The comparison between the outcome of the endoscopy and histopathology outcome is graphically given in Figure 1, but we have added another Supplementary table 1 with the exact numbers in detail.

Finally, a further discussion is needed with regards to the AI tools for the confirmation of these cases. For example, I would suggest the authors take into account and discuss the following recent work: "A digital pathology workflow for the segmentation and classification of gastric glands: Study of gastric atrophy and intestinal metaplasia cases".

- We agree AI is the future to optimize endoscopist and pathologist performance and we have added a section covering this including the mentioned reference in the discussion (page 17, 14 -20).

  Generally, I think that this article will certainly be of interest to many readers of PLOS One.
